# A Transcriptome Array-Based Approach Links Proteinuria and Distinct Molecular Signatures to Intrarenal Expression of Type I Interferon *IFNA5* in Lupus Nephritis

**DOI:** 10.3390/ijms241310636

**Published:** 2023-06-25

**Authors:** Peter Korsten, Björn Tampe

**Affiliations:** Department of Nephrology and Rheumatology, University Medical Center Göttingen, 37075 Göttingen, Germany; peter.korsten@med.uni-goettingen.de

**Keywords:** lupus nephritis, type I interferon, *IFNA5*, proteinuria

## Abstract

In systemic lupus erythematosus (SLE), the relevance of non-hematopoietic sources of type I interferon in human autoimmunity has recently been recognized. Particularly, type I interferon production precedes autoimmunity in early skin lesions related to SLE. However, the relevance of intrarenal type I interferon expression has not been shown in lupus nephritis. From transcriptome array datasets, median-centered log_2_ mRNA expression levels of IFNα (*IFNA1*, *IFNA2*, *IFNA4*, *IFNA5*, *IFNA6*, *IFNA7*, *IFNA8*, *IFNA10*, *IFNA13*, *IFNA14*, *IFNA16*, *IFNA17*, and *IFNA21*), IFNω (*IFNW1*), and IFNβ (*IFNB1*) in lupus nephritis were extracted specifically from microdissected tubulointerstitial (*n* = 32) and glomerular compartments (*n* = 32). We found an association between proteinuria and tubulointerstitial expression of type I interferon *IFNA5* (*p* = 0.0142), while all others were not significantly associated. By contrast, no such correlation was observed between proteinuria and any type I interferon expression in the glomerular compartment in lupus nephritis. Interestingly, there was no difference between female and male patients (*p* = 0.8237) and no association between type I interferon *IFNA5* expression and kidney function or lupus nephritis progression. Finally, we identified distinct molecular signatures involved in transcriptional regulation (GLI protein-regulated transcription, IRF7 activation, and HSF1-dependent transactivation) and receptor signaling (BMP signaling and GPCR ligand binding) in association with tubulointerstitial expression of type I interferon *IFNA5* in the kidney. In summary, this transcriptome array-based approach links proteinuria to the tubulointerstitial expression of type I interferon *IFNA5* in lupus nephritis. Because type I interferon receptor subunit I antagonism has recently been investigated in active SLE, the current study further emphasizes the role of type I interferons in lupus nephritis and might also be of relevance for mechanistic studies.

## 1. Introduction

Systemic lupus erythematosus (SLE) is a chronic, multisystem autoimmune disease characterized by reoccurring flares and remissions [1]. Clinical manifestations of SLE vary in severity and presentation, including life-threatening renal and neuropsychiatric morbidity in addition to pathogenic secondary complications [2]. Multiple genetic and environmental factors trigger SLE in predisposed individuals, contributing to the complex epidemiology of affected populations [3]. An important serological hallmark of SLE is the presence of autoantibodies that are capable to bind to circulating nuclear antigens. A strong association also exists between the presence of anti-double-stranded DNA (anti-dsDNA), anti-Ro and anti-ribonucleoprotein, and type I interferon activity in SLE [4,5]. A growing body of evidence supports the roles of type I interferons in the immunopathogenesis of SLE and other interferonopathies [6,7,8]. Given the key role of type I interferons in the initiation and perpetuation of autoimmunity in SLE, many efforts have been made to obtain an in-depth understanding of type I interferons and the development of targeted SLE therapies through intervening in IFN signaling pathways [9].

The family of type I interferons includes 13 interferon (IFN)-α subtypes, IFN-β, IFN-δ, IFN-ε, IFN-κ, and IFN-ω. Type I interferons and the IFN-α receptors 1 (IFNAR1) and 2 (IFNAR2) form a functional IFNAR complex, leading to tyrosine phosphorylation of signal transducers and activation of transcription 1 (STAT1) and 2 (STAT2) [10,11]. Phosphorylated STAT1 and STAT2 translocate with interferon regulatory factor 9 (IRF9) to the nucleus and drive IFN-stimulated response element (ISRE) activation. ISRE promotes transcription of multiple interferon-responsive genes, leading to the production of multiple proinflammatory and immunomodulatory factors involved in the host innate immunity in response to viral infection [12,13]. IFNAR activation also mediates cell-intrinsic induction of ISRE to produce type I interferons, thereby amplifying the interferon-driven response [14,15,16]. Thus, immune cells can rapidly produce large amounts of IFN at localized sites that ultimately can lead to tissue injury and disease exacerbation [17]. Multiple genetic polymorphisms increase type I interferon signaling and are associated with increased susceptibility to SLE [18]. Increased type I interferon expression and downstream type I interferon-induced molecular signatures correlate with SLE severity, and therapeutic use of type I interferons for patients with viral hepatitis can induce a lupus-like syndrome [19,20,21]. The primary source of type I interferons appears to be plasmacytoid dendritic cells (pDCs) that are capable to incorporate immune complexes containing DNA/RNA through Fcγ receptor (FcγR) IIA (CD32A). In turn, DNA/RNA uptake activates intracellular nucleic acid-sensing toll-like receptors (TLRs) that trigger the induction of type I interferons [22,23].

Recently, the relevance of non-hematopoietic sources of type I interferons in human autoimmunity has also been recognized in SLE [24]. Particularly, type I interferon production precedes autoimmunity in early skin lesions related to systemic lupus erythematosus [24]. Furthermore, it was recently hypothesized that this local type I interferon synthesis is also relevant for lupus nephritis [25]. Autoimmune connective tissue diseases are recognized to arise from asymptomatic preclinical autoimmunity. Autoantibodies precede symptoms by years and are far more common than clinical autoimmune diseases [26,27,28,29]. Hence, autoantibody-positive individuals constitute a population at risk of whom only a minority will develop clinical autoimmunity. A key determinant of progression to overt autoimmune disease is the level of interferon activity [26]. In humans, type I interferons have been described predominantly in active class IV lupus nephritis [30]. Moreover, expression of type I interferon has been observed in renal proximal tubular epithelial cells (RPTECs) and tubulointerstitial synthesis in human class IV lupus nephritis [30].

However, the relevance of local synthesis and intrarenal expression of type I interferons regarding distinct molecular signatures has not been shown in lupus nephritis. Therefore, we here pursued a transcriptome array-based approach to dissect the relevance of intrarenal type I interferon expression in lupus nephritis. 

## 2. Results

**Figure 1:**
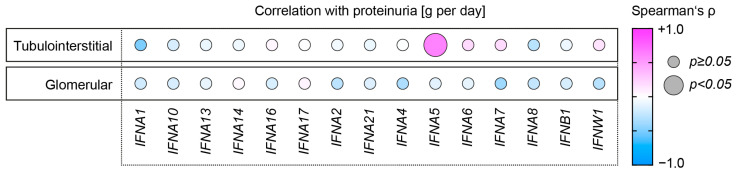
Proteinuria is associated with intrarenal expression of type I interferon *IFNA2* in lupus nephritis. Median-centered log_2_ mRNA expression levels of IFNα (*IFNA1*, *IFNA2*, *IFNA4*, *IFNA5*, *IFNA6*, *IFNA7*, *IFNA8*, *IFNA10*, *IFNA13*, *IFNA14*, *IFNA16*, *IFNA17*, and *IFNA21*), IFNω (*IFNW1*), and IFNβ (*IFNB1*) in microdissected tubulointerstitial (*n* = 32) and glomerular compartments (*n* = 32) are shown in correlation with proteinuria in lupus nephritis. The heatmap reflects the mean values of Spearman’s ρ; circle size represents the significance level.

Because proteinuria is most sensitive for early renal involvement and outcomes in SLE, we first compared tubulointerstitial (*n* = 32) and glomerular (*n* = 32) type I interferon mRNA expression levels available from transcriptome array datasets (*IFNA1*, *IFNA2*, *IFNA4*, *IFNA5*, *IFNA6*, *IFNA7*, *IFNA8*, *IFNA10*, *IFNA13*, *IFNA14*, *IFNA16*, *IFNA17*, and *IFNA21*), IFN-ω (*IFNW1*), and IFN-β (*IFNB1*) in lupus nephritis with proteinuria in lupus nephritis (Supplementary Tables S1–S3) [31,32,33,34]. Specifically, we identified a positive association between proteinuria and tubulointerstitial expression of type I interferon *IFNA5* (*p* = 0.0142), while all other type I interferons were not significantly associated (Figure 1). However, there was no correlation between proteinuria and any type I interferon expression in the glomerular compartment in lupus nephritis (Figure 1). In summary, proteinuria was associated with intrarenal expression of type I interferon *IFNA5* in lupus nephritis.

**Figure 2:**
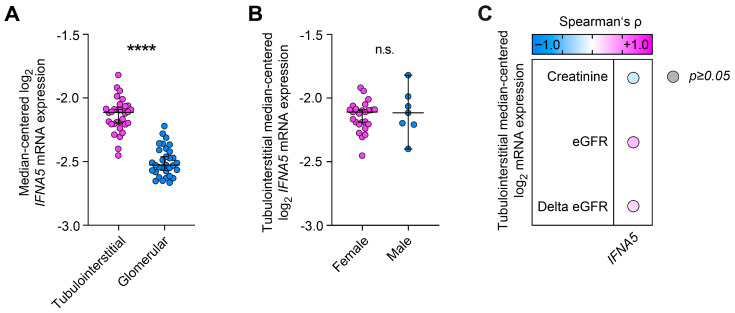
Predominant tubulointerstitial type I interferon *IFNA5* expression is independent of sex, kidney function at time of kidney biopsy, or kidney function thereafter (delta eGFR) in lupus nephritis. (**A**) Median-centered log_2_ *IFNA5* mRNA expression levels in lupus nephritis are shown by group separation for tubulointerstitial and glomerular mRNA expression levels. Group comparisons were performed using the Mann–Whitney U-test to determine differences in medians (**** *p* < 0.0001). (**B**) Tubulointerstitial median-centered log_2_ *IFNA5* mRNA expression levels in lupus nephritis are shown by group separation for female and male sex. Comparisons of groups were performed using the Mann–Whitney U-test to determine differences in medians (n.s. not significant). (**C**) Correlations between tubulointerstitial *IFNA5* mRNA expression levels and laboratory markers of kidney function in lupus nephritis are shown by heatmap reflecting the mean values of Spearman’s ρ; the circle size represents the significance level.

Next, we characterized tubulointerstitial and glomerular type I interferon *IFNA5* expression with regard to clinical and laboratory parameters in lupus nephritis. We identified a predominant tubulointerstitial expression of *IFNA5* compared to the glomerular compartment (*p* < 0.0001, Figure 2A). Moreover, there was no difference between female and male patients (*p* = 0.8237, Figure 2B) or any association between type I interferon *IFNA5* expression, kidney function reflected by serum creatinine, and estimated glomerular filtration rate (eGFR) at the time of biopsy or thereafter (delta eGFR) in lupus nephritis (Figure 2C). In summary, this transcriptome array-based approach links proteinuria to the tubulointerstitial expression of type I interferon *IFNA5* in lupus nephritis.

**Figure 3:**
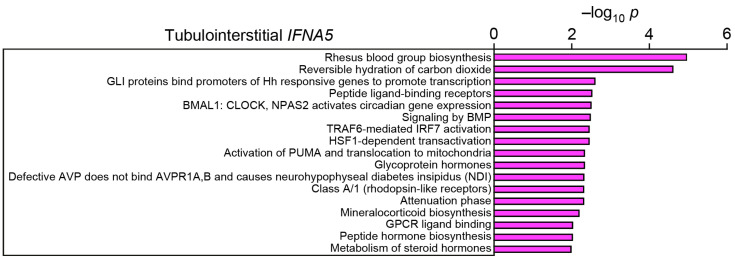
Tubulointerstitial type I interferon *IFNA5* expression is associated with distinct molecular signatures in the kidney: −log_10_ *p* values of signaling pathways associated with tubulointerstitial *IFNA5* mRNA expression in the kidney are shown.

Finally, we aimed to identify molecular signatures associated with tubulointerstitial expression of type I interferon *IFNA5*. Tubulointerstitial *IFNA5* expression was associated with enrichment of distinct molecular signatures. Particularly, pathways were enriched that are involved in transcriptional regulation: glioma-associated oncogene transcription factor (GLI) protein-regulated transcription, interferon regulatory factor 7 (IRF7) activation, and heat shock factor 1 (HSF1)-dependent transactivation (Figure 3 and Supplementary Table S4). Moreover, several receptor signaling pathways were associated with intrarenal *IFN5A* expression: bone morphogenic protein (BMP) signaling and G-protein coupled receptor (GPCR) ligand binding (Figure 3 and Supplementary Table S4). In summary, we here identified novel molecular signatures associated with intrarenal expression of type I interferon *IFNA5*. 

## 3. Discussion

Interferons are potent stimulators of autoimmunity in a wide range of tissues affected by SLE, including the vasculature, and all nucleated cells express type I interferon receptors. Most tissues affected by SLE exhibit higher expression of interferon-stimulated genes than healthy individuals. Accumulating evidence indicates that tissue sources of type I interferons in response to environmental or other local stimuli are relevant in disease pathogenesis and clinical presentation rather than circulating sources [24,25]. Here we pursued a transcriptome array-based approach and linked proteinuria to the intrarenal expression of type I interferon *IFNA5* in lupus nephritis.

Genetic studies have established a positive association between *IFNA5* and SLE [35]. Regarding the kidney, it has already been shown that type I interferons are highly expressed in tubular epithelial cells [30]. In lupus nephritis, it has been demonstrated that local production of IFN-α was associated with a type I interferon signature in renal proximal tubular epithelium [30]. Therefore, local synthesis of type I interferon may act with an autocrine effect on RPTECs, leading to amplification of the tubulointerstitial injury in lupus nephritis. This aligns with our observation that *IFNA5* is predominantly expressed in the tubulointerstitial compartment in lupus nephritis. Stimulation of tubular epithelial cells with type I interferon induces the expression of the immunoproteasome subunit LMP-7 that might shape the repertoire of antigens presented via MHC class I molecules [30]. Similarly, upregulation of LMP-7 is found in lupus nephritis along with transcriptional induction of gene sets involved in antigen processing and presentation, response to hypoxia, and SLE pathogenesis [30]. Consistently, results from single-cell transcriptomic studies in lupus nephritis demonstrated that tubular epithelial cell interferon scores are associated with proliferative histology, markers of fibrosis, a keratinocyte interferon score, and non-response to therapy [36]. By contrast, interferon scores in B, T, and NK cells and in monocytes are not associated with clinical response. In addition, the number of dendritic cells is low in lupus nephritis and is not associated with interferon-stimulated genes [36]. These observations suggest non-hematopoietic local sources of type I interferon in human SLE autoimmunity [24,25].

We here provide further insights into the intrarenal expression of type I interferons and show that tubulointerstitial *IFNA5* expression might be relevant specifically in lupus nephritis autoimmunity. Furthermore, there was no association between type I interferon *IFNA5* expression and kidney function or progressive disease. These observations imply that type I interferon *IFNA5* expression might be of relevance, particularly in early stages of renal involvement in SLE, which is also of relevance for outcome [31,32]. Finally, we identified distinct molecular signatures associated with intrarenal expression of type I interferon *IFNA5*. Among them, these were involved in transcriptional regulation (GLI protein-regulated transcription, IRF7 activation, and HSF1-dependent transactivation) and receptor signaling (BMP signaling and GPCR ligand binding). Interestingly, a functional variant of *IRF7* with consecutive activation has already been shown to predispose to the development of SLE [37]. Furthermore, we here identified an association between intrarenal *IFNA5* expression and GPCR ligand binding. As part of the GPCR protein family, the complement receptor C5aR (synonym CD88) has already been described in human lupus nephritis [38,39]. Moreover, enhanced C5a/C5aR signaling has been shown to promote development of experimental lupus nephritis [40]. These observations support our finding that identified molecular signatures associated with intrarenal type I interferon could be of relevance, requiring further investigation especially in the context of current therapeutic strategies targeting interferon signaling in lupus nephritis.

Among emerging therapeutic approaches targeting interferon signaling, type I interferon receptor subunit I antagonism by anifrolumab has recently been investigated in active SLE [41]. Anifrolumab (also known as MEDI546) is a fully human, effector-null, Ig G1 κ monoclonal antibody that binds to IFNAR1 and thereby blocks type I interferon signaling [41]. Anifrolumab is currently in clinical development for treatment of SLE and lupus nephritis. Its crystal structure confirmed that anifrolumab sterically inhibits type I interferons by binding to IFNAR1 [42]. Anifrolumab was engineered with a triple mutation L234F/L235E/P331S in the heavy chain to reduce engagement with the cell surface receptor FcγR and potential Fc-mediated effector functions (e.g., effector-null), such as antibody-dependent cell-mediated cytotoxicity and complement-dependent cytotoxicity. Therefore, the current study further emphasizes the role of type I interferons in lupus nephritis and might also be of relevance for mechanistic studies. We also identified that proteinuria correlated with intrarenal synthesis of complement C5. Moreover, this is of particular interest since ongoing trials are currently testing efficacy and safety of anti-C5 antibodies (clinical trial: NCT04564339) and C5a receptor (C5aR) antagonists (clinical trial: NCT02151409) in patients with lupus nephritis.

Although we are aware that these observations require further validation in experimental models, we here provide the first evidence to link proteinuria and intrarenal expression of type I interferon *IFNA5* in lupus nephritis.

## 4. Materials and Methods

### 4.1. Analyses of Publicly Available Array Datasets

Publicly available datasets were extracted from the Nephroseq database (www.nephroseq.org, 30 April 2023, University of Michigan, Ann Arbor, MI, USA) [34]. Particularly, median-centered log_2_ mRNA expression levels of *IFNA1* (reporter ID: 208375_at), *IFNA2* (211338_at), *IFNA4* (207964_x_at), *IFNA5* (214569_at), *IFNA6* (208548_at), *IFNA7* (208259_x_at), *IFNA8* (207932_at), *IFNA10* (208261_x_at), *IFNA13* (208344_x_at), *IFNA14* (208182_x_at), *IFNA16* (208448_x_at), *IFNA17* (211405_x_at), *IFNA21* (211145_x_at), *IFNW1* (207817_at), and *IFNB1* (208173_at) were extracted specifically from microdissected tubulointerstitial (*n* = 32) and glomerular compartments (*n* = 32, platform: Affymetrix Human Genome U133 Plus 2.0 Array, altCDF v10). 

### 4.2. Gene Set Enrichment

For gene set enrichment analysis, genes coexpressed with *IFNA5* were extracted from the whole dataset (including 199 glomerular and 201 tubulointerstitial compartments) from the Nephroseq database (www.nephroseq.org, 30 April 2023, University of Michigan, Ann Arbor, MI, USA). Pathway analysis was performed for gene enrichment associated with log_2_ *IFNA5* mRNA expression with a correlation threshold of ≥0.4 by using reactome (http://reactome.org, 30 April 2023); pathways with a predefined entities value of *p* ≤ 0.01 were included and shown in Supplementary Table S4.

### 4.3. Statistical Analysis

Spearman’s correlation was used to assess the correlation between continuous variables, and the Mann–Whitney U-test was used to determine differences between medians. Data analyses were performed with GraphPad Prism (version 9.3.1 for macOS, GraphPad Software, San Diego, CA, USA). A value of *p* < 0.05 was considered statistically significant.

## Data Availability

The original contributions presented in the study are included in the article’s Supplementary Materials; further data and material are available from the corresponding author upon reasonable request.

## Supplementary Materials

The supporting information can be downloaded at: https://www.mdpi.com/article/10.3390/ijms241310636/s1.
