# Supplementary material for: A Transcriptome Array-Based Approach Links Proteinuria and Distinct Molecular Signatures to Intrarenal Expression of Type I Interferon IFNA5 in Lupus Nephritis
Source: Int J Mol Sci. 2023 Jun 25;24(13):10636. doi: 10.3390/ijms241310636 (PMC10341446; doi:10.3390/ijms241310636)
Supplement: Supplementary file 1 [file ijms-24-10636-s001.zip › ijms-2423201-supplementary.pdf]

A Transcriptome Array-Based Approach Links Proteinuria and Distinct Molecular Signatures to Intrarenal Expression of Type I Interferon *IFNA5* in Lupus Nephritis

Supplementary Material

Peter Korsten and Björn Tampe *

Department of Nephrology and Rheumatology, University Medical Center Göttingen,

37075 Göttingen, Germany; peter.korsten@med.uni-goettingen.de

* Correspondence: bjoern.tampe@med.uni-goettingen.de; Tel.: +49-5513910575

**Table S1. Clinical and laboratory parameters of included samples extracted from the Nephroseq database.**				
**Sample**	**Sex**	**Serum creatinine level (mg/dL)**	**eGFR (MDRD)**	**Delta eGFR**
LN301_Tub	Female	0,9	75	2,324202117
LN302_Tub	Female	0,7	101	4,958448515
LN304_Tub	Male	0,9	94	49,96546067
LN305_Tub	Male	0,9	97	-1,612261619
LN306_Tub	Female	1,1	62	4,459519742
LN307_Tub	Female	1,1	61	3,086092528
LN308_Tub	Female	3,2	17	-0,210183092
LN309_Tub	Female	1	75	37,10504736
LN310_Tub	Female	1,6	37	4,621487095
LN311_Tub	Male	1,3	58	-1,880480943
LN312_Tub	Female	1,6	38	6,955256086
LN313_Tub	Female	3,1	19	-2,377861108
LN314_Tub	Female	1,2	51	3,488368579
LN318_Tub	Female	3	20	-0,776793162
LN319_Tub	Female	2,2	31	11,07525153
LN323_Tub	Female	0,7	104	-3,141816823
LN329_Tub	Female	0,78	102	-5,001560199
LN336_Tub	Female	3,7	14	
LN338_Tub	Female	0,9	73	-4,744832513
LN340_Tub	Female	1,1	54	-11,01588705
LN342_Tub	Female	0,6		
LN343_Tub	Female	1,8	38	10,95524633
LN346_Tub	Female			
LN348_Tub	Male	1,2	72	2,127400855
LN349_Tub	Male	1	99	
LN352_Tub	Female	2,7	22	
LN357_Tub	Female	1,16	63	14,98655473
LN358_Tub	Male	1,17	83	-7,985578763
LN359_Tub	Female	0,75	97	-2,284700347
LN360_Tub	Female	1,03	68	7,789398888
LN361_Tub	Male	0,98	93	1,894894043
LN362_Tub	Female	0,84	93	-1,998540763

Table S2. Included samples and corresponding log_2 mRNA expression levels extracted from the Nephroseq database for tubulointerstitial mRNA expression levels of complement components.

Sample	IFNA1 / 208375_at	IFNA10 / 208261_x_at	IFNA13 / 208344_x_at	IFNA14 / 208182_x_at	IFNA16 / 208448_x_at	IFNA17 / 211405_x_at	IFNA2 / 211338_at	IFNA21 / 211145_x_at	IFNA4 / 207964_x_at	IFNA5 / 214569_at	IFNA6 / 208548_at	IFNA7 / 208259_x_at	IFNA8 / 207932_at	IFNB1 / 208173_at	IFNW1 / 207817_at
LN301_Tub	-2,08047	-1,65372	0,27475	-1,17219	-1,98604	-0,48206	-2,28553	-0,91387	-1,30563	-2,04937	-2,06165	-1,16509	-2,36714	-1,66683	-1,23954
LN302_Tub	-1,97323	-1,76888	0,55028	-1,31693	-1,84488	-0,24964	-2,19791	-0,89311	-1,21963	-2,18881	-1,72601	-0,97061	-2,29868	-1,31557	-1,41559
LN304_Tub	-2,05384	-1,90176	0,37865	-1,55699	-1,91631	-0,56298	-2,2115	-0,99934	-1,26676	-2,39952	-1,73944	-1,22182	-2,27097	-1,12142	-1,27352
LN305_Tub	-2,06502	-1,60073	0,35504	-1,05436	-1,7828	-0,37023	-1,90816	-0,69549	-1,09117	-2,11573	-1,85127	-0,6471	-2,12856	-1,1831	-1,45649
LN306_Tub	-1,96779	-1,50934	0,38123	-1,35917	-1,6836	-0,39711	-2,20255	-0,88988	-1,2627	-2,09268	-1,93797	-0,65541	-2,2819	-1,85533	-1,0994
LN307_Tub	-2,05293	-1,54591	0,5811	-1,12715	-1,60481	-0,26186	-2,32218	-0,85792	-1,0248	-1,91794	-1,76175	-0,87257	-2,3622	-1,37816	-1,24369
LN308_Tub	-2,0959	-1,77156	0,14102	-1,47803	-1,7542	-0,59652	-2,31233	-1,08268	-1,31884	-2,45246	-1,89239	-1,01483	-2,54103	-1,81787	-1,25897
LN309_Tub	-1,96978	-1,64407	0,3697	-1,27018	-1,80119	-0,37938	-2,24318	-0,98237	-1,10933	-2,00947	-1,81279	-1,00878	-2,4031	-1,28603	-1,14552
LN310_Tub	-2,05713	-1,78435	0,20882	-1,24005	-1,883	-0,70345	-2,1458	-1,1233	-1,34412	-2,18925	-1,83617	-1,19855	-2,4315	-1,53191	-1,39674
LN311_Tub	-2,01192	-1,4979	0,39485	-1,20485	-1,69591	-0,38055	-2,0334	-1,12039	-1,16074	-2,20852	-2,0334	-0,96848	-2,3632	-1,57608	-1,16898
LN312_Tub	-2,24018	-1,67533	0,26602	-1,43895	-1,85579	-0,27596	-2,29562	-0,86516	-1,2609	-2,2385	-1,90895	-1,15364	-2,39213	-1,57422	-1,31254
LN313_Tub	-2,15523	-1,4989	0,55586	-1,29599	-1,84568	-0,34825	-2,26979	-0,85342	-1,20595	-2,18548	-1,89356	-1,04342	-2,4103	-1,5943	-1,37023
LN314_Tub	-2,25216	-1,47094	0,61906	-0,99384	-1,76791	-0,10114	-2,31867	-0,91618	-1,1559	-2,06038	-1,7235	-1,11694	-2,26927	-1,39865	-1,16944
LN318_Tub	-2,0718	-1,5846	0,40579	-1,12511	-1,52343	-0,44873	-2,15224	-0,82657	-0,82351	-2,16599	-1,93128	-0,75365	-2,32195	-1,5753	-1,42301
LN319_Tub	-2,06198	-1,36599	0,5637	-1,11837	-1,67178	-0,14947	-2,24966	-0,85826	-1,111	-2,09772	-1,83654	-0,91337	-2,31859	-1,41361	-1,49886
LN323_Tub	-1,8347	-1,56096	0,57893	-1,09862	-1,81971	0,02787	-2,33987	-0,92505	-1,01931	-2,08949	-1,90873	-0,82507	-1,96842	-1,27674	-1,07847
LN329_Tub	-2,27407	-1,45433	0,2601	-1,25135	-1,92308	-0,44391	-2,09877	-0,90578	-1,26594	-2,10895	-1,95747	-1,23747	-2,50931	-1,56133	-1,3212
LN336_Tub	-2,20765	-1,63176	0,57234	-1,21398	-1,68664	-0,30099	-2,08658	-0,99141	-1,11728	-2,20461	-1,90793	-1,00034	-2,34365	-1,60677	-1,35997
LN338_Tub	-2,1496	-1,71705	0,07373	-1,16546	-1,8411	-0,66175	-2,18113	-1,17907	-1,30976	-2,0859	-2,02742	-1,17482	-2,22587	-1,72802	-1,45746
LN340_Tub	-2,0481	-1,74892	0,22276	-1,47866	-1,96811	-0,67498	-2,28516	-1,13035	-1,28337	-2,20582	-2,08848	-1,20573	-2,37004	-1,59998	-1,37607
LN342_Tub	-2,02493	-1,74314	0,39821	-1,4871	-1,78404	-0,58792	-2,16452	-1,20556	-1,2068	-2,30553	-2,06158	-1,08489	-2,34494	-1,57125	-1,44668
LN343_Tub	-2,19222	-1,67923	0,18536	-1,36731	-1,89108	-0,61268	-2,27154	-1,16664	-1,2932	-2,09217	-2,00635	-1,37431	-2,50055	-1,71711	-1,41548
LN346_Tub	-2,18651	-1,75543	0,19551	-1,24194	-1,95464	-0,40872	-2,16086	-0,86126	-1,10954	-2,28846	-1,63264	-1,06929	-2,31158	-1,14118	-1,42799
LN348_Tub	-2,07301	-1,57797	0,76007	-1,3739	-1,5916	-0,39316	-2,22638	-0,97279	-1,14278	-1,8191	-1,9423	-0,86296	-2,39581	-1,53814	-1,19483
LN349_Tub	-2,18834	-1,62125	0,37985	-1,16103	-1,72898	-0,2632	-2,38491	-1,06997	-1,16232	-1,98653	-1,92614	-0,93014	-2,33245	-1,33688	-1,4212
LN352_Tub	-2,13748	-1,63244	0,33697	-1,23159	-1,6904	-0,53405	-2,35958	-1,15149	-1,26872	-2,2741	-2,09915	-1,15513	-2,23846	-1,52875	-1,35104
LN357_Tub	-2,11307	-1,58782	0,40083	-1,63712	-1,74331	-0,28115	-2,18682	-0,94055	-1,29045	-1,94301	-1,84002	-1,41478	-2,32803	-1,47318	-1,09078
LN358_Tub	-1,95207	-1,54166	0,4094	-1,18161	-1,77083	-0,33416	-2,23322	-1,01292	-1,19758	-2,19681	-1,85298	-1,12585	-2,42855	-1,45475	-1,36203
LN359_Tub	-2,07771	-1,51579	0,36791	-1,11983	-1,72739	-0,36188	-2,22385	-1,10291	-1,10214	-2,1205	-1,96475	-1,01188	-2,40965	-1,68954	-1,3179
LN360_Tub	-2,19632	-1,80639	0,36226	-1,23981	-1,69272	-0,39823	-2,20746	-1,24811	-1,20249	-2,0969	-1,98046	-0,92112	-2,36354	-1,39857	-1,34374
LN361_Tub	-2,06726	-1,66582	0,22592	-1,07396	-1,85468	-0,32695	-2,27562	-0,90233	-1,08878	-2,0648	-1,5771	-1,04041	-2,31626	-1,64322	-1,1518
LN362_Tub	-2,15535	-1,6665	0,30013	-1,41174	-1,89745	-0,49387	-2,31984	-1,24954	-1,51271	-2,10991	-1,95309	-1,11137	-2,34137	-1,56522	-1,34532

Table S3. Included samples and corresponding log_2 mRNA expression levels extracted from the Nephroseq database for glomerular mRNA expression levels of complement components.

Sample	IFNA1 / 208375_at	IFNA10 / 208261_x_at	IFNA13 / 208344_x_at	IFNA14 / 208182_x_at	IFNA16 / 208448_x_at	IFNA17 / 211405_x_at	IFNA2 / 211338_at	IFNA21 / 211145_x_at	IFNA4 / 207964_x_at	IFNA5 / 214569_at	IFNA6 / 208548_at	IFNA7 / 208259_x_at	IFNA8 / 207932_at	IFNB1 / 208173_at	IFNW1 / 207817_at
LN401_Glom	-2,50451	-1,73995	-0,56132	-1,85801	-1,97893	-0,74454	-2,49545	-1,25245	-1,51307	-2,57571	-2,27257	-1,14337	-2,56138	-1,80049	-1,3134
LN402_Glom	-2,45326	-1,67562	-0,41558	-1,61672	-1,81682	-0,4403	-2,27861	-1,30735	-1,48491	-2,47016	-2,41277	-0,96116	-2,52619	-1,70005	-1,51747
LN404_Glom	-2,5055	-1,42683	-0,2322	-1,62742	-2,07116	-0,52922	-2,43053	-1,11098	-1,37703	-2,35942	-2,37637	-1,22576	-2,49287	-1,92371	-1,23857
LN405_Glom	-2,36916	-1,43245	-0,46002	-1,55626	-1,65265	-0,35678	-2,32439	-1,20443	-1,17571	-2,48789	-2,34374	-0,95766	-2,74022	-1,43551	-1,29854
LN406_Glom	-2,30793	-1,57524	-0,41309	-1,81795	-2,05704	-0,8546	-2,38654	-1,32863	-1,49821	-2,35655	-2,4698	-1,33965	-2,5592	-1,61178	-1,03867
LN407_Glom	-2,41777	-1,58516	-0,64042	-2,01108	-2,0704	-0,58068	-2,4702	-1,50986	-1,50051	-2,62936	-2,39606	-1,19576	-2,45981	-2,00039	-1,2077
LN408_Glom	-2,45235	-1,67425	-0,51328	-1,82703	-2,03033	-0,67655	-2,57346	-1,23973	-1,54614	-2,52627	-2,35953	-1,35791	-2,61289	-1,89479	-1,48244
LN409_Glom	-2,39875	-1,59463	-0,57988	-1,66655	-1,89128	-0,47193	-2,37285	-1,20118	-1,33897	-2,50848	-2,19352	-1,31101	-2,4818	-1,81485	-1,21429
LN410_Glom	-2,53403	-1,73827	-0,59291	-1,68766	-2,1693	-0,58631	-2,46085	-1,19171	-1,47804	-2,63053	-2,43066	-1,42543	-2,6809	-1,70239	-1,18473
LN411_Glom	-2,3341	-1,67354	-0,33735	-1,41875	-1,81378	-0,36272	-2,51379	-0,95995	-1,14137	-2,39757	-2,31056	-1,06675	-2,59247	-1,86096	-1,28915
LN412_Glom	-2,4675	-1,27459	-0,18412	-1,2799	-1,49329	-0,15056	-2,39153	-0,91656	-1,27769	-2,31168	-2,40074	-1,14085	-2,61922	-1,68494	-1,23574
LN413_Glom	-2,40138	-2,0145	-0,50958	-1,86994	-2,09091	-0,7303	-2,53878	-1,27192	-1,71916	-2,61383	-2,48315	-1,29426	-2,7545	-1,66745	-1,18077
LN414_Glom	-2,36377	-1,56422	-0,72221	-1,77985	-1,92653	-0,67527	-2,42455	-1,28844	-1,4596	-2,66563	-2,35586	-1,06162	-2,38	-1,88537	-1,09708
LN418_Glom	-2,5507	-1,54791	-0,39187	-1,57864	-1,75823	-0,4064	-2,51622	-1,14095	-1,42896	-2,54435	-2,38203	-1,21604	-2,6524	-1,5651	-1,22015
LN419_Glom	-2,40057	-1,59762	-0,3911	-1,77668	-2,02803	-0,48868	-2,4632	-1,36559	-1,55719	-2,54906	-2,3618	-1,25008	-2,58565	-1,82632	-1,20783
LN423_Glom	-2,26479	-1,47177	-0,52829	-1,80501	-1,9125	-0,78547	-2,41977	-1,02906	-1,51102	-2,65295	-2,23239	-1,21659	-2,45066	-1,65204	-0,89501
LN430_Glom	-2,15875	-1,23968	0,09943	-1,23216	-1,35322	-0,25189	-2,34525	-0,81748	-1,08603	-2,2802	-2,23384	-0,78841	-2,55047	-1,41568	-1,28592
LN438_Glom	-2,30384	-1,72988	-0,33367	-1,94481	-2,11377	-0,80284	-2,60642	-1,21895	-1,43515	-2,47365	-2,33592	-1,4053	-2,67249	-1,85786	-1,34993
LN440_Glom	-2,37176	-1,36946	-0,07553	-1,59178	-1,79532	-0,45097	-2,49182	-0,98879	-1,29988	-2,40883	-2,2873	-0,92404	-2,45276	-1,20429	-1,09183
LN442_Glom	-2,35647	-1,47896	-0,39856	-1,59366	-1,70781	-0,56839	-2,42524	-1,10735	-1,29348	-2,22103	-2,38107	-1,10189	-2,5422	-1,48621	-1,2385
LN444_Glom	-2,31597	-1,55373	-0,58804	-1,81064	-2,13859	-0,6715	-2,41693	-1,15732	-1,08946	-2,52994	-2,4005	-1,07769	-2,56087	-1,71096	-1,295
LN445_Glom	-2,4103	-1,61631	-0,36329	-2,04907	-1,8717	-0,74407	-2,44911	-1,21694	-1,59253	-2,58225	-2,37407	-1,35068	-2,46387	-1,56336	-1,17368
LN448_Glom	-2,42439	-1,53189	-0,36731	-1,67289	-2,05836	-0,72717	-2,61405	-1,12189	-1,39382	-2,36635	-2,204	-1,36951	-2,55806	-1,69798	-1,01675
LN450_Glom	-2,35296	-1,8507	-0,59033	-1,83289	-2,15588	-0,75272	-2,30968	-1,08172	-1,37242	-2,46157	-2,26913	-1,39638	-2,67857	-2,03796	-1,10112
LN451_Glom	-2,29781	-1,57875	-0,08399	-1,95421	-2,00188	-0,6017	-2,31989	-1,19695	-1,37446	-2,56956	-2,33428	-0,96226	-2,53558	-1,58948	-1,02521
LN454_Glom	-2,30391	-1,66011	-0,58012	-1,90976	-1,94629	-0,5283	-2,52659	-1,40355	-1,34868	-2,51105	-2,32021	-1,10077	-2,44932	-1,60553	-1,2863
LN462_Glom	-2,42907	-1,58342	-0,57325	-1,89571	-2,12417	-0,8109	-2,56835	-1,32777	-1,51948	-2,62439	-2,48698	-1,54229	-2,66474	-1,86598	-1,28766
LN463_Glom	-2,46748	-1,57268	-0,45767	-1,76169	-1,9451	-0,67057	-2,61254	-1,3734	-1,43734	-2,54416	-2,38071	-1,33043	-2,62745	-1,64422	-1,29502
LN464_Glom	-2,37709	-1,68571	-0,24545	-1,84287	-1,96599	-0,81272	-2,67896	-1,25596	-1,48426	-2,56445	-2,40378	-1,20896	-2,60125	-1,67527	-1,3577
LN465_Glom	-2,4985	-1,54055	-0,42717	-1,75672	-1,93003	-0,66184	-2,44352	-1,30127	-1,34706	-2,42245	-2,28944	-1,1568	-2,58315	-1,88545	-1,15167
LN466_Glom	-2,56579	-1,75499	-0,44952	-1,91813	-2,02014	-0,70028	-2,56645	-1,14991	-1,33426	-2,52798	-2,37005	-1,15532	-2,68272	-1,61685	-1,29934
LN467_Glom	-2,48847	-1,52716	-0,50498	-1,83176	-2,07684	-0,62341	-2,49818	-1,29549	-1,34892	-2,6501	-2,49699	-1,19678	-2,68417	-1,86751	-1,4434

**Table S4. Gene set enrichment in association with intrarenal *IFNA5* expression.**			
**Pathway name**	**reactome identifier**	***p value***	**Species**
Rhesus blood group biosynthesis	R-HSA-9037628	1,00E-5	Homo sapiens
Reversible hydration of carbon dioxide	R-HSA-1475029	2.22E-5	Homo sapiens
GLI proteins bind promoters of Hh responsive genes to promote transcription	R-HSA-5635851	2.31E-3	Homo sapiens
Peptide ligand-binding receptors	R-HSA-375276	2.76E-3	Homo sapiens
BMAL1: CLOCK, NPAS2 activates circadian gene expression	R-HSA-1368108	2.9E-3	Homo sapiens
Signaling by BMP	R-HSA-201451	3.05E-3	Homo sapiens
TRAF6-mediated IRF7 activation	R-HSA-933541	3.25E-3	Homo sapiens
HSF1-dependent transactivation	R-HSA-3371571	3.27E-3	Homo sapiens
Activation of PUMA and translocation to mitochondria	R-HSA-139915	4.3E-3	Homo sapiens
Glycoprotein hormones	R-HSA-209822	4.3E-3	Homo sapiens
Defective AVP does not bind AVPR1A,B and causes neurohypophyseal diabetes insipidus (NDI)	R-HSA-5619099	4.32E-3	Homo sapiens
Class A/1 (rhodopsin-like receptors)	R-HSA-373076	4.45E-3	Homo sapiens
Attenuation phase	R-HSA-3371568	4.46E-3	Homo sapiens
Mineralocorticoid biosynthesis	R-HSA-193993	5.84E-3	Homo sapiens
GPCR ligand binding	R-HSA-500792	8.51E-3	Homo sapiens
Peptide hormone biosynthesis	R-HSA-209952	8.8E-3	Homo sapiens
Metabolism of steroid hormones	R-HSA-196071	9.31E-3	Homo sapiens
